# Use of Resveratrol Self-Emulsifying Systems in T/C28a2 Cell Line as Beneficial Effectors in Cellular Uptake and Protection Against Oxidative Stress-Mediated Death

**DOI:** 10.3389/fphar.2018.00538

**Published:** 2018-05-24

**Authors:** Solenn Le Clanche, Tristan Cheminel, François Rannou, Dominique Bonnefont-Rousselot, Didier Borderie, Christine Charrueau

**Affiliations:** ^1^UMR-S 1124 INSERM Toxicologie, Pharmacologie et Signalisation Cellulaire, CUSP, Sorbonne Paris Cité, Université Paris Descartes, Paris, France; ^2^Unité Pédagogique de Biochimie, Faculté de Pharmacie de Paris, Sorbonne Paris Cité, Université Paris Descartes, Paris, France; ^3^Service de Rééducation et de Réadaptation de l’Appareil Locomoteur et des Pathologies du Rachis, Hôpital Cochin (AP-HP), Paris, France; ^4^Service de Biochimie Métabolique, Hôpitaux Universitaires Pitié-Salpêtrière-Charles Foix (AP-HP), Paris, France; ^5^INSERM U 1022 CNRS UMR 8258, Chimie ParisTech, PSL Research University, Unité de Technologies Chimiques et Biologiques pour la Santé, Faculté de Pharmacie de Paris, Sorbonne Paris Cité, Université Paris Descartes, Paris, France; ^6^Service de Diagnostic Biologique Automatisé, Hôpital Cochin (AP-HP), Paris, France

**Keywords:** anti-oxidative activity, chondrocyte, osteoarthritis, nano-emulsion, resveratrol

## Abstract

Osteoarthritis (OA) is the most prevalent rheumatic disease in the world. Although its etiology is still unknown, one of the key processes in OA progression and development is oxidative stress. In this context, resveratrol, a well-known anti-oxidant from the stilbene family, could be of particular interest in future OA therapeutic strategies. However, currently, because of its low bioavailability, use of resveratrol in human health is very limited. In this study, we tested two resveratrol self-emulsifying systems previously developed in our laboratory in order to determine if they could improve cellular uptake of resveratrol in a human immortalized chondrocytic cell line (T/C28a2) and enhance protection against oxidative stress. Our results showed that resveratrol self-emulsifying systems were able first to increase cellular tolerance towards resveratrol, and thus decrease resveratrol intrinsic cellular toxicity, allowing the use of higher concentrations, second, to increase resveratrol uptake in membrane and intracellular fractions, and finally, to improve protection against oxidative stress-mediated death in human immortalized chondrocytic cell line T/C28a2. These data suggest that new formulations of resveratrol could be considered as potential beneficial effectors in future OA treatments.

**Chemical compounds cited in this article:** Resveratrol (PubChem CID: 445154); isopropyl myristate (PubChem CID: 8042); Polysorbate 80 (PubChem CID: 5284448); ethanol (PubChem CID: 702); Medium-chain triglycerides (PubChem CID: 93356).

## Introduction

Osteoarthritis is a degenerative disease of the joint, characterized by irreversible cartilage degradation. It is the most prevalent rheumatic disease in the world (Muirden, 2005; Vos et al., 2012), generally associated with aging, making it a real major health issue. Currently, its etiology is still unclear but it is known that it is the result of an association of several factors (mechanical, genetic, metabolic, cellular, and biochemical) leading to a progressive and irreversible cartilage degradation (Goldring and Marcu, 2009). Under physiological conditions, articular cartilage is a specific, avascular, and aneural tissue, composed by a unique cell type, chondrocytes, which are responsible for the extra-cellular matrix (ECM) components synthesis.

Oxidative stress is one of the key processes in OA development, as an increase in reactive oxygen species (hydrogen peroxide, nitric oxide, etc.) production is responsible for chondrocyte dysfunction and apoptosis, leading to cartilage destruction (Asada et al., 2001; Sutipornpalangkul et al., 2009; Poulet and Beier, 2016). Chondrocyte apoptosis is a severe event in OA progression because these cells have a very low turn-over so their loss implies an important decrease in ECM components synthesis, leading to a general failure of the articular cartilage (Hwang and Kim, 2015). Despite progress made in the understanding of the different mechanisms involved in OA, no effective disease-modifying treatment has been discovered yet (Kim et al., 2018). Consequently, the development of new therapeutic approaches able to stop OA progression and development could potentially avoid resorting to surgery. The importance of tuning ROS levels in chondrocytes is highlighted in a recent study by Sun et al. (2017) who designated regulators of redox states as potential therapeutic targets in OA. Among promising molecules, resveratrol (3,5,4′-trihydroxystilbene), a polyphenol from the stilbene family well-known for its multiple beneficial effects *in vitro* (including antioxidant, anti-inflammatory, anti-diabetic properties (Smoliga et al., 2011)) could be of particular interest. Indeed, protective effects of resveratrol in OA have been demonstrated in some studies. Dave et al. (2008) have shown that resveratrol was able to protect chondrocytes from IL-1β-induced apoptosis by inhibition of mitochondrial membrane depolarization in OA chondrocytes *in vitro.* Besides, in an experimental rabbit model of OA, Wang et al. (2012) have demonstrated that resveratrol was responsible for a decrease in cartilage destruction, chondrocyte apoptosis, and a reduction of overproduced nitric oxide in synovial fluid.

However, resveratrol bioavailability is very limited because of its fast metabolism and degradation *in vivo*. Indeed, in humans, the oral consumption of resveratrol (25 mg/kg) leads to only 1.7 to 1.9% of free form of resveratrol found in the peak serum concentrations (Goldberg et al., 2003). In order to overcome this bioavailability problem, current therapeutic strategies are now turned towards development of new formulations of resveratrol like nanoparticles (Jose et al., 2014; Karthikeyan et al., 2015), liposomes (Csiszár et al., 2015) or self-emulsifying drug delivery systems (SEDDS) (Sessa et al., 2014; Yutani et al., 2014), potentially allowing for an increase in its efficacy in human health by increasing its stability, solubility or capacity to cross cellular membrane.

In this study, we chose to use SEDDS generating nano-emulsions (NE) of resveratrol, previously developed in our laboratory, which demonstrated increased antioxidant properties in endothelial cells (Amri et al., 2014). We carried out experiments on an immortalized human chondrocytic cell line (T/C28a2) possessing the original morphology and appropriate phenotypic responses of joint chondrocytes, as well as high proliferative activity (Goldring et al., 1994).

The aim of this study was to determine whether the use of resveratrol NE generated from SEDDS dispersion could increase cellular uptake of resveratrol in order to improve its effects on chondrocytes submitted to high oxidative stress, as seen in OA development. These new formulations, designed to be injectable intra-articularly, could overcome resveratrol intestinal and hepatic metabolism observed after oral administration, which is responsible for a decrease in the amount of resveratrol reaching target organs.

## Materials and Methods

### Reagents

All reagents used for cell culture were from Sigma-Aldrich (Saint-Quentin-Fallavier, France). Reagents and chemicals used for other experiments are specified in each section. To avoid any isomerization of *trans*-resveratrol into *cis*-resveratrol, all the resveratrol formulations were protected from light at each step of preparation and testing.

### Formulation of the SEDDS

*Trans*-resveratrol was purchased from Sigma-Aldrich (Saint-Quentin-Fallavier, France). Medium-chain triglycerides (Miglyol^®^ 812) were purchased from Sasol (Witten, Germany), polysorbate 80 (Montanox^®^ 80 VG PHA) from Seppic (Paris, France), and isopropyl myristate and ethanol 96% v/v from Cooper (Melun, France).

Two SEDDS previously formulated to enhance both cellular uptake and antioxidant efficacy of resveratrol were tested (Amri et al., 2014). Briefly, SEDDS were composed as follows : 20% isopropyl myristate, 70% Montanox^®^ 80, and 10% ethanol for SEDDS 1; 20% Miglyol^®^ 812, 70% Montanox^®^ 80, and 10% ethanol for SEDDS 2. The nanoemulsions (NE) resulting from the self-emulsification of the SEDDS in the cell culture medium were characterized by a droplet size of 24 ± 7 and 103 ± 14 nm (Polydispersity Index 0.291 ± 0.062, and 0.389 ± 0.051) and a zeta potential of -15.8 ± 2.6 and -14.7 ± 2.0 mV, for NE 1 and NE 2, respectively. The NE, either free or containing 25 and 50 μM of resveratrol, were evaluated in cytotoxicity and antioxidant assays.

### Cell Culture

Immortalized human T/C28a2 chondrocytes were kindly provided by M. B. Goldring’s laboratory (Hospital for Special Surgery, Weill Cornell Medicine, New York, NY, United States). Briefly, chondrocytes isolated from the human costal cartilage from a 15-year-old female were transfected using the simian virus 40 large T antigen (SV40-TAg). These cells present the classical characteristics of human chondrocytes (Goldring et al., 1994).

Cells were cultivated in 75-cm^2^ flasks or in 96-well plates, in DMEM/Ham’s F12 (v/v) medium (5 mM glucose) supplemented with 10% fetal bovine serum (FBS), 1% antibiotics (penicillin/streptomycin) at 37°C in a humidified 5% CO_2_ incubator until they reached 80% confluence.

Cells were used between passages 3 and 10.

All treatments were made in DMEM/Ham’s F12 (v/v) supplemented with 1% FBS, 1% antibiotics (penicillin/ streptomycin) and 1% ethanol (Sigma-Aldrich, Saint-Quentin-Fallavier, France), as previously published (Notas et al., 2006; Frombaum et al., 2011; Frombaum et al., 2012; Amri et al., 2014). Besides, a neutral red assay was performed to evaluate effects of 1% ethanol on the chondrocytic cell line viability. No difference in viability was observed between cells incubated with media supplemented with 1% ethanol and cells incubated with media without ethanol (data not shown).

### Evaluation of Resveratrol Formulation Effect on Cell Viability

In order to evaluate the possible toxicity of the two resveratrol formulations on the chondrocytic cell line, a neutral red assay was performed. A stock solution of 50 mM resveratrol was prepared in 100% ethanol. Cells were incubated with NE 1 and 2 and with resveratrol in ethanolic solution (1% Ethanol final concentration in culture medium), at both concentrations (25 and 50 μM), in a 96-well plate for 24 h at 37°C in a humidified 5% CO_2_ incubator. The plate was emptied by reversal before 100 μL of a neutral red solution (Sigma-Aldrich, Saint-Quentin-Fallavier, France) was added to each well. Cells were then incubated for 3 h at 37°C in a humidified 5% CO_2_ incubator. Afterwards, the plate was emptied and 100 μL of a formol–calcium solution (37% formaldehyde, 0.1 g/mL calcium chloride dihydrate; Sigma-Aldrich, Saint-Quentin-Fallavier, France) were added. After 1 min of incubation, the plate was emptied and each well received 100 μL of a solution of ethanol (50% v/v) and pure acetic acid (1% v/v) (Sigma-Aldrich, Saint-Quentin-Fallavier, France). After gently mixing the plate for 5 min, absorbance at 540 nm was read on a microplate reader (MultiskanEx^®^, Thermo Electron Corporation, Asnières-sur-Seine, France). Results were expressed as mean viability percentage ± SEM (*n* = 5).

Cell viability above 95% (total innocuity threshold) was required for further experiments.

Nomenclature used for each experimental condition is detailed in **Table 1**.

**Table 1:** Definition of nomenclature used for all experimental conditions.

Nomenclature	Experimental condition
Unloaded NE	Nano-emulsion without resveratrol
NE 25 μM Res	Nano-emulsion with 25 μM of resveratrol
NE 50 μM Res	Nano-emulsion with 50 μM of resveratrol

### Determination of Intracellular and Membrane Concentrations of Resveratrol in T/C28a2 Cells

Cells were incubated in 75-cm^2^ flasks with the selected resveratrol formulations, i.e., resveratrol 25 μM in 1% ethanol, NE 1 and 2 with resveratrol 25 or 50 μM, for 20, 40, 60, and 180 min. Then, cells were washed with ice-cold PBS and trypsinized in order to collect cell lysates. Pellets were washed twice with ice-cold PBS by centrifugation for 10 min, at 1500 *g*, at 4°C and then harvested into 0.4 mL of lysis buffer supplemented with 5% proteases inhibitors. Finally, protein concentrations were determined in cell lysates using a Bradford assay kit (Bio-Rad, Marnes-la-Coquette, France).

Measurement of resveratrol concentrations in membrane and intracellular compartments were performed by HPLC as previously described (Frombaum et al., 2012). Briefly, membrane and intracellular fractions were isolated by centrifugation of cell lysates (15 min at 6000 *g*). Then, 100 μL of methanol (Sigma-Aldrich, Saint-Quentin-Fallavier, France) were added to the supernatant containing the intra-cellular fraction, and 200 μL of mobile phase (distilled water/methanol (v/v), with 0.5% acetic acid) were added to the pellet containing the membrane fraction. After centrifugation for 10 min at 2500 *g*, 100 μL of supernatant of each fraction were injected in the column. Resveratrol concentrations in each cellular compartment were expressed as means ± SEM in nmol/mg of proteins (*n* = 4).

### Evaluation of Resveratrol Cytoprotective Effect Against Oxidative Stress-Mediated Death

The effect of the two resveratrol formulations against oxidative stress-mediated death was evaluated thanks to cell viability assessment. Cells were pre-incubated in a 96-well plate overnight either with the selected resveratrol formulations (NE 1 and NE 2 at 25 and 50 μM resveratrol) in culture medium, or with unloaded NE 1 and NE 2 in culture medium, or with resveratrol 25 μM in 1% ethanol in culture medium, or with the conventional unsupplemented medium. Then, the plate was emptied before cells were further incubated either with different concentrations of H_2_O_2_ (200, 500 and 750 μM) or co-incubated with these H_2_O_2_ concentrations and resveratrol in the selected formulations, for 24 h at 37°C in a humidified 5% CO_2_ incubator. Then, a neutral red assay was performed as described above (Section “Evaluation of Resveratrol Formulation Effect on Cell Viability”). Results were expressed as mean viability percentage ± SEM (*n* = 4).

Control cells were cultured in conventional medium and incubated with neither resveratrol nor H_2_O_2_.

### Statistical Analysis

Results were expressed as means ± SEM. All statistical analyses were performed with GraphPad Prism 5©, using a Kruskal–Wallis test. Data were considered statistically significant at *p* < 0.05.

## Results

### Cytotoxicity Assay

**FIGURE 1:**
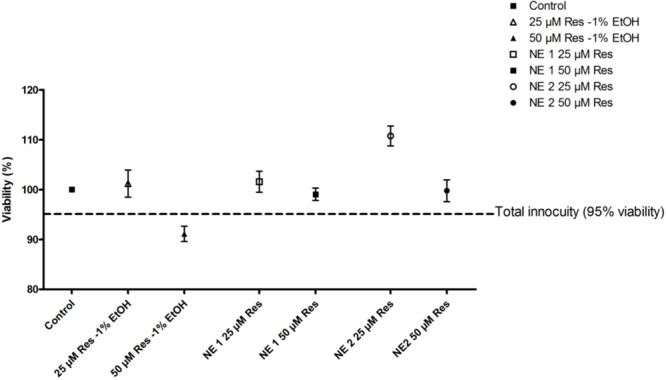
Viability of cells incubated with the resveratrol nano-emulsions or resveratrol in ethanolic solution vs. unexposed control cells. Results are expressed as mean viability percentage ± SEM (*n* = 5).

NE 1 and 2 at 25 and 50 μM resveratrol, and 25 μM resveratrol in ethanolic solution induced no change in cell viability in the chondrocytic cell line after incubation for 24 h. Nevertheless, 50 μM resveratrol in the ethanolic solution induced a cell viability of 91%, thus under the total innocuity threshold defined at 95%. Consequently, this concentration of resveratrol in the ethanolic solution was no longer studied (**Figure 1**).

### Measurement of Resveratrol Concentrations in Membrane and Intra-Cellular Fractions

**FIGURE 2:**
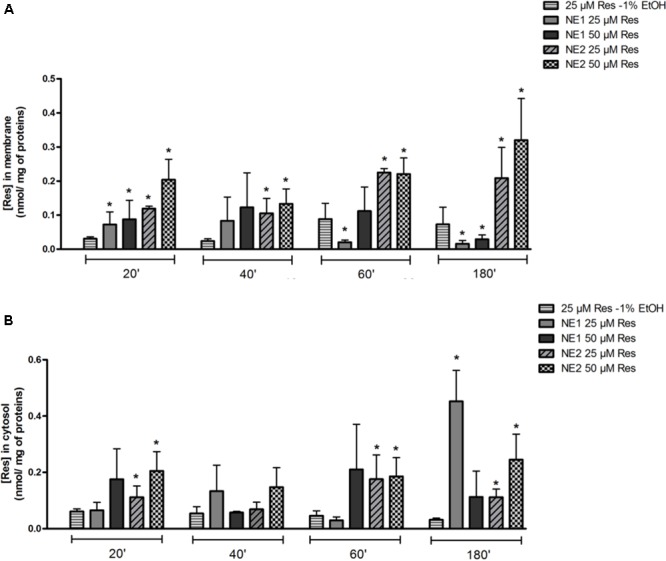
Measurement of resveratrol concentrations in membrane **(A)** and intra-cellular **(B)** fractions by HPLC in the chondrocytic cell line T/C28a2. Cells were incubated either with the resveratrol nano-emulsions (25 or 50 μM) or resveratrol in ethanolic solution (25 μM) for 20, 40, 60, and 180 min. Results are expressed as mean ± SEM in nmol/mg of protein (*n* = 4). ^∗^*p* < 0.05 vs. resveratrol (25 μM) in ethanolic solution.

In the membrane fraction, 25 or 50 μM of resveratrol brought by NE 2 allowed a significant increase in resveratrol concentration at any time of incubation compared with 25 μM of resveratrol as an ethanolic solution (*p* < 0.05). When 25 μM or 50 μM of resveratrol was brought by NE 1, a significant increase in resveratrol concentration was observed after 20 min of incubation (*p* < 0.05 vs. 25 μM resveratrol-1% ethanol), followed by a significant decrease at 60 (25 μM) and 180 min (25 and 50 μM) (*p* < 0.05 vs. 25 μM resveratrol-1% ethanol) (**Figure 2A**).

In the intracellular fraction, 25 μM of resveratrol in NE 1 induced a significant increase in resveratrol concentration after 180 min (*p* < 0.05 vs. 25 μM resveratrol-1% ethanol). 25 and 50 μM of resveratrol brought by NE 2 induced a significant increase in resveratrol concentrations after incubation for 20, 60, and 180 min compared with 25 μM resveratrol-1% ethanol (*p* < 0.05) (**Figure 2B**).

### Evaluation of Resveratrol Cytoprotective Effect Against Oxidative Stress-Mediated Death

**FIGURE 3:**
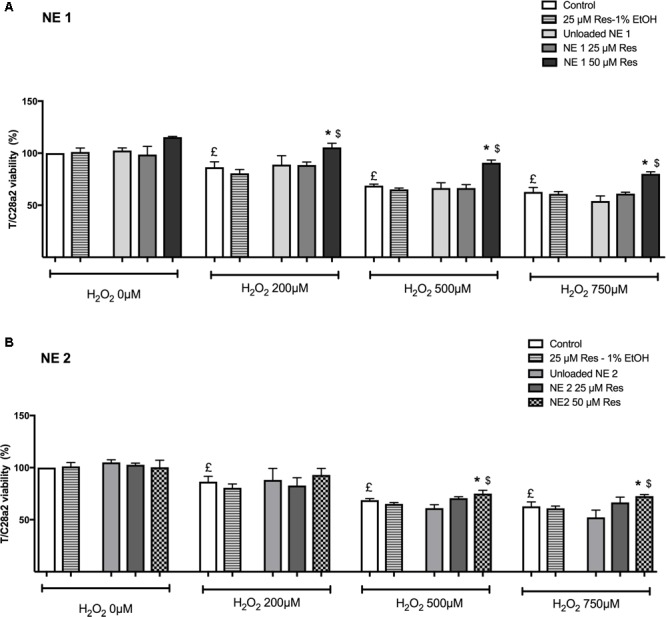
Effect of resveratrol either in the nano-emulsions (**A**: NE1; **B**: NE2) or in the ethanolic solution against oxidative stress-mediated death. Results were expressed as mean viability percentage ± SEM (*n* = 4). Control cells were cultured in conventional medium and neither incubated with resveratrol nor H_2_O_2_. Unloaded NE were also evaluated. ^£^*p* < 0.05 vs. control cells incubated neither with resveratrol nor H_2_O_2_. ^∗^*p* < 0.05 vs. cells incubated with H_2_O_2_ without resveratrol. ^$^*p* < 0.05 vs. cells incubated with H_2_O_2_ and resveratrol (25 μM) in ethanolic solution.

As expected in the cellular model of oxidative stress, incubation for 24 h with H_2_O_2_ (200, 500, and 750 μM) induced a significant decrease in cell viability whatever H_2_O_2_ concentration (*p* < 0.05 vs. control cells) (**Figure 3**). Of note, resveratrol at 50 μM in 1% ethanol was not tested due to its cytotoxic effect.

In the NE 1 panel (**Figure 3A**), 50 μM of resveratrol were able to protect cells against oxidative stress-mediated death at each H_2_O_2_ concentration (*p* < 0.05 vs. all other groups).

In the NE 2 panel (**Figure 3B**), 50 μM of resveratrol allowed an increased cell viability after incubation with 500 and 750 μM H_2_O_2_ compared with control cells and cells incubated with 25 μM of resveratrol in ethanolic solution (*p* < 0.05).

In addition, 50 μM of resveratrol brought by NE 1 was more efficient than brought by NE 2 after incubation with 500 μM H_2_O_2_ (*p* < 0.05), but no significant difference was observed after incubation with 200 and 750 μM H_2_O_2_ (**Table 2**).

**Table 2:** Comparison of protective effect of 50 μM of resveratrol in NE 1 and NE 2 on cell viability, after incubation with 200, 500, and 750 μM H_2_O_2_.

[H_2_O_2_] (μM)	NE 1	NE 2	*P* value
**200**	105.5 ± 7.9 %	92.9 ± 8.4 %	ns
**500**	90.7 ± 4.5 %	75.1 ± 5.5 %	*P* < 0.05
**750**	80.1 ± 3.4 %	72.7 ± 2.5 %	ns

## Discussion

Osteoarthritis is the first rheumatic disease in the world and the main cause of disability in the older population, making it a major public health issue as no curative treatment currently exists to stop its progression and development. Resveratrol, a polyphenol from the stilbene family, is known for its multiple beneficial properties like antioxidant, anti-diabetic and anti-inflammatory activities. Some *in vitro* studies have shown a beneficial effect of resveratrol in OA. Dave et al. (2008) have demonstrated that incubation of primary human osteoarthritic chondrocytes with resveratrol prior to stimulation with IL-1β had several beneficial effects. Indeed, it was firstly responsible for a decrease in matrix metalloprotease (MMP) 1, 3, and 13 synthesis, which are catabolic enzymes mainly involved in cartilage degradation. Secondly, it induced an increase in proteoglycan production allowing reducing OA development, and finally, resveratrol was responsible for an inhibition of mitochondrial membrane depolarization which is involved in chondrocyte apoptosis process. In 2008, Shakibaei et al. (2008) have demonstrated that resveratrol was able to inhibit apoptosis in human chondrocytes by inhibition of NF-κB signaling pathway. Furthermore, in a rabbit model of OA, intra-articular injection of resveratrol was able to decrease cartilage degradation and protect the joint from OA development (Elmali et al., 2005). These data suggest that resveratrol could be of particular interest in future human OA structural therapeutic strategies. However, currently, because of its low bioavailability, use of resveratrol in human health is very limited.

In order to overcome this bioavailability issue, numerous new formulations of resveratrol are under development (Amri et al., 2012). One of them is based on the use of SEDDS responsible for the generation of nano-emulsions, which is the promising approach we chose in the present study.

Although commonly tested on various cultured cells, resveratrol could present deleterious effects on cell viability, especially when used at elevated concentrations as previously demonstrated by several studies (Juhasz et al., 2010; Amri et al., 2014; Risuleo, 2016). As an example, Kucinska et al. (2014) showed in Jurkat cell line that resveratrol concentrations above 25 μM were cytotoxic. Interestingly, in the present study on the human chondrocytic cell line T/C28a2, both NE tested allowed providing cells with 25 and 50 μM of resveratrol without toxicity, while resveratrol at 50 μM under classic formulation (1% EtOH final concentration) induced a cell viability under the total innocuity threshold (95% viability). Hence, by decreasing resveratrol intrinsic toxicity, both NE allowed to increase the concentration of resveratrol tested in T/C28a2 cell line.

Once safe NE and resveratrol concentrations were defined on the T/C28a2 cell line, the cellular distribution of resveratrol between membrane and intracellular compartments was determined. NE 1 led to a significant increase of resveratrol at the earliest time of 20 min in cell membrane, and at the latest time of 180 min in the cytosol. A significant increase was observed in both membrane and cytosol localizations with NE 2 at all times compared with resveratrol 25 μM-1% EtOH. Resveratrol concentration brought by NE 2 was rather stable in the cytosol whereas it increased with time in the membrane compartment. These results suggest that resveratrol brought by NE 2 remained essentially localized inside the membrane instead of reaching the cytosol. On the other hand, after an early increase, resveratrol concentration brought by NE 1 progressively decreased in the membrane compartment while an important increase occurred in the cytosol at the latest incubation time, suggesting a transfer of resveratrol from the cellular membrane toward the cytosol. This different behavior between both NE could result from differences in their composition and droplet size characteristics. As for composition parameter, the oily phases used in SEDDS formulations were not the same as SEDDS 1 contained isopropyl myristate, and SEDDS 2 contained Miglyol^®^ 812, making them potentially more or less lipophilic. Miglyol^®^ 812 is composed of medium-chain triglycerides while isopropyl myristate is an ester of isopropanol and tetradecanoic acid. It is known that triglycerides are able to interact with the phospholipidic bilayer of biological membranes (especially with phosphatidylcholine), as well as phospholipid-rich surface monolayers of plasma lipoproteins, in a highly specific way (Hamilton, 1989). This kind of interactions could favor the incorporation of NE into cell membranes and slow down the crossing of the NE through the phospholipidic bilayer; an early release of resveratrol inside the plasma membrane through the affinity of the NE droplets with the membrane components could be hypothesized. Concerning the droplet size parameter, i.e. 24 ± 7 nm for NE 1 vs. 103 ± 14 nm for NE 2, it could play a key role in the crossing of cellular membranes, since larger droplets of NE 2 could be slowed down by the phospholipidic bilayer and membrane proteins.

**FIGURE 4:**
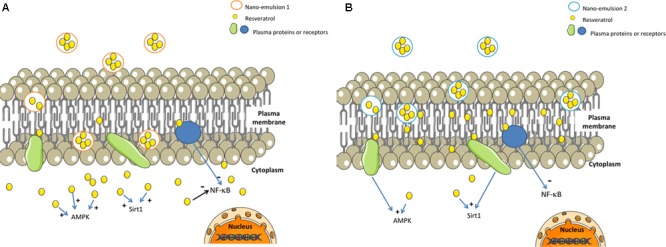
Possible mechanisms of action of resveratrol-loaded NE 1 and NE 2. AMPK, adenosine monophosphate activated protein kinase; Res, resveratrol; Sirt 1, silent mating type information regulation 2 homolog 1. Resveratrol brought in NE 1 was able to cross the cellular membrane and to reach the cytoplasm **(A)**, whereas resveratrol brought by NE 2 was mainly localized inside the plasma membrane **(B)**. Both resveratrol-loaded NE were able to protect cells against oxidative stress-mediated death.

However, despite its main localization in membranes, resveratrol brought by NE 2 was able to protect chondrocytes against cell death mediated by H_2_O_2_, in a nearly similar level as NE 1. We hypothesize that these results could be partly explained by the capacity of resveratrol to act as a signal transducer in cell membranes, thus interacting with several receptors or proteins responsible for activation of downstream signaling pathways involved in different cellular mechanisms (Bastianetto et al., 2009). Resveratrol brought by NE 1 was essentially found in the intra-cellular compartment so that one could hypothesize that its cytoprotective effect against oxidative stress would be due to direct interactions with several effectors of intra-cellular signaling pathways like adenosine monophosphate activated protein kinase (AMPK) (Breen et al., 2008; Hou et al., 2008), silent mating type information regulation 2 homolog 1 (Sirt 1) (Howitz et al., 2003), or NF-κB (Pendurthi et al., 1999; Ashikawa et al., 2002) (**Figure 4**).

Currently, no efficient treatment for OA has been discovered to stop OA progression and avoid surgery, so the only therapeutic strategies available are focused on symptomatic relief of pain. Unfortunately, long-term use of non-steroidal anti-inflammatory drugs (NSAIDs) is responsible for severe damages on the gastrointestinal tract (Lanas et al., 2015). When patients are non-responsive or intolerant to NSAIDs, a viscosupplementation consisting in intra-articular injection of hyaluronic acid can be proposed (Migliore and Procopio, 2015). The interest of such a treatment has been discussed, and according to both the Osteoarthritis Research Society International 2012 guideline and the American College of Rheumatology 2013 guidelines, hyaluronic acid treatment was neither recommended nor discouraged. However, it has shown many benefits such as intra-articular lubrication, anti-inflammatory, analgesic and chondroprotective effects, among others. The recent analysis of literature reporting clinical studies that evaluated hyaluronic acid-based therapy for OA leads to nuanced conclusions: Bowman et al. (2018) underlined a significant pain relief, and Altman et al. (2018) concluded that repeated courses of intra-articular hyaluronic acid injections allowed maintaining or further improving pain reduction while introducing no increased safety risk. In this context, the development of injectable resveratrol nano-emulsions which could overcome the low oral bioavailability and could be used intra-articularly alone, or combined with hyaluronic acid, depending on the stage of the disease, seems promising to protect chondrocytes from oxidative damages and to slow down OA progression and development.

In conclusion, our study suggests that nano-emulsions of resveratrol could be considered as beneficial effectors in future OA therapeutic strategies, notably by increasing the concentration of resveratrol reaching the target cells and by limiting the deleterious effect of oxidative stress on chondrocytes.

## Author Contributions

SLC, CC, DB-R, DB, and FR contributed to the conception and design of the study. SLC, TC, and CC carried out the experiments. SLC performed the statistical analysis and wrote the first draft of the manuscript. SLC and CC wrote sections of the manuscript. All authors contributed to manuscript revision and read and approved the submitted version.

## Conflict of Interest Statement

CC is the guest editor of Frontiers research topic “Future opportunities for resveratrol delivery.” The other authors declare that the research was conducted in the absence of any commercial or financial relationships that could be construed as a potential conflict of interest.

## Abbreviations

ECM: extra-cellular matrix
NE: nano-emulsion
NSAIDs: non-steroidal anti-inflammatory drugs
OA: osteoarthritis
SEDDS: self-emulsifying drug delivery system
